# Health technology assessment criteria as drivers of coverage with managed entry agreements: a case study of cancer medicines in four countries

**DOI:** 10.1007/s10198-022-01526-x

**Published:** 2022-10-11

**Authors:** Olina Efthymiadou

**Affiliations:** Medical Technology Research Group, Department of Health Policy, London School of Economics, Houghton Street, London, WC2A 2AE England

**Keywords:** Managed entry agreements, HTA decision-making, Conditional reimbursement, Risk-sharing, Discounts

## Abstract

**Background:**

Managed entry agreements (MEAs) continue to emerge in health technology assessment (HTA)-based decision-making, to address evidentiary uncertainties arising therein. Evidence on the HTA criteria that influence MEAs' uptake remains scarce. This study explores the HTA criteria that determine (i) if an HTA funding decision will be listed with conditions (LWC) other than a MEA, or with a MEA as a condition (LWCMEA), and ii) the MEA type implemented (i.e., financial, outcomes based, or combination).

**Methods:**

HTA reports of all oncology medicines approved since 2009 in Australia, England, Scotland, and Sweden were searched to capture the clinical/economic evidence uncertainties raised in the decision-making process, the Social Value Judgements (SVJs) considered therein and the final coverage decision. Binary and multinomial logit models captured the probability (odds ratio (OR)) of a coverage decision being LWCMEA vs. LWC, and of the MEA being financial, outcomes based, or combination, based on the HTA criteria studied.

**Results:**

23 (12%) LWC and 163 (88%) LWCMEA decisions were identified; 136 (83.4%) comprised financial, 10 (6.2%) outcomes based and 17 (10.4%) combination MEAs. LWCMEA decisions were driven by economic model utilities' uncertainties (7.16 < *OR* < 26.7, *p* < .05), and the innovation (8.5 < *OR* < 11.7, *p* < .05) SVJ. Outcomes based contracts were influenced by clinical evidence (*OR* = 69.2, *p* < .05) and relevance to clinical practice (*OR* = 26.4, *p* < .05) uncertainties, and rarity (*OR* = 46.2, *p* < .05) and severity (*OR* = 23.3, *p* < .05) SVJs. Financial MEAs were influenced by innovation (8.9 < *OR* < 9.3, *p* < .05) and societal impact (*OR* = 17.7, *p* < .0001) SVJs.

**Conclusions:**

This study provides an empirical framework on the HTA criteria that shape payers' preferences in funding with MEAs, when faced with uncertainty.

**Supplementary Information:**

The online version contains supplementary material available at 10.1007/s10198-022-01526-x.

## Background and objectives

The rapid progress of therapeutic innovation and the respective introduction of new, high-cost, therapies might be favourable from the patient’s perspective, but from the payer’s perspective, it poses challenges in managing the market entry and long-term affordability of these therapies [1]. To mitigate these pressures, countries are developing policies to facilitate decision-making about the reimbursement of novel, high-cost pharmaceuticals, such as the cost-effectiveness appraisal of these technologies. In many countries worldwide, these evaluations take the form of health technology assessment (HTA), a process where the clinical and cost-effectiveness of these products is assessed by national competent authorities, to understand if these products demonstrate the “value-for-money” profile required by different healthcare systems to enable coverage [2, 3]. In the HTA process, challenges may arise due to evidentiary uncertainties generated by the immature or early phase evidence submitted by manufactures for appraisal. The uncertainties facing decision-makers have been classified into three broader categories including (i) clinical (e.g., the applicability of study endpoints and treatment population to the actual clinical practice in the country of interest), (ii) financial (e.g., the actual number of doses and treatment duration required in real-world practice and the respective aggregate budget impact) and (iii) utilisation uncertainties (e.g., the appropriate prescribing of the product for the patient population in which it is deemed to be cost-effective) [2, 4]. To address uncertainties arising in the HTA-based decision-making, managed entry agreements (MEAs) between payers and manufacturers are increasingly being employed in many countries as part of the process. Depending on the type of uncertainty to be addressed, literature has classified MEAs in two broader categories, namely outcome- and financial-based agreements depending on whether they aim to mitigate uncertainties related to drug performance or not respectively, while combination agreements with financial and outcome-based aspects have also been observed [2, 5, 6]. Literature has shown that even in cases where countries implemented a MEA for the same medicine-indication pair, often presenting with similar uncertainties, there was still variation in the types of agreements implemented and the respective objective targeted by these agreements [7–9]. Descriptive studies have suggested that health system-specific considerations and perceptions of “risk” across settings might play a role in explaining such differences [4, 8, 10]. Furthermore, a descriptive, comparative analysis of MEAs for cancer medicines in different settings, found that cross-country differences may arise chiefly due to payers’ preferences on social value considerations, such as the socioeconomic and Quality of Life (QoL) impact of the treatment appraised, followed by setting-specific requirements on the economic model, and the comparators, costs, and utilities included in the model [11].

Despite the growing utilisation of MEAs, quantitative, empirical research on the HTA factors that have an impact on the uptake of MEAs across settings remains scarce [12, 13]. This has significant implications for the transparency of “best-practice” guidelines on MEA negotiation and implementation processes across and within countries [6, 13]. To address this literature gap, this paper provides a quantitative assessment of the key HTA criteria that have been suggested by previous, descriptive research as potential determinants of MEAs. Ultimately, the objective of this analysis is twofold: first, to identify the relative importance of these criteria in comparison to each other towards shaping decision-making under uncertainty and second, using specifically a quantitative approach, to map the HTA criteria that determine coverage with a MEA or not, and if so, the criteria that determine the type of MEA.

## Methods

### Sample selection

All oncology medicines which obtained regulatory approval by the European Medicines Agency (EMA) in Europe and by the Therapeutic Goods Administration (TGA) in Australia between 1st January 2009 and 15th June 2018 (at the medicine-indication pair level) were studied in Australia (AUS), England (ENG), Scotland (SCOT) and Sweden (SE). Oncology was selected as the study therapeutic class because it has been documented to be the therapeutic class with the largest proportion of implemented MEAs and the therapeutic class where MEAs continue to be increasingly implemented [2]. Study countries were selected because they all implement MEAs, they all have long-established HTA policies and processes to guide their coverage decisions, they have both a publicly available list of MEAs and HTA reports (or publicly available documents where MEAs and other HTA criteria can be inferred from, such as the Public Summary Documents in Australia) and they use similar approaches in their pricing and reimbursement decision-making process (i.e., clinical and cost-effectiveness approach), allowing for comparability across agencies [14].

### Data collection

The conceptual framework underpinning data collection operates under the overarching hypothesis that HTA coverage decisions (whether positive, negative or restricted) are primarily shaped by the HTA process itself, including the evidence appraised therein (whether clinical, economic or otherwise), the way this evidence is interpreted/assessed by the decision-makers, and the broader socioeconomic and political context in which the decision-making takes place [11, 14, 15].

Essentially, this framework divides the HTA process and relevant variables of interest in four “buckets” where it is hypothesised that a combination of variables within “buckets” (A), (B) and (C), determine the observed outcome in “bucket” (D) as follows: (A) clinical and economic evidence appraised (e.g., trial characteristics, comparators, Incremental Cost Effectiveness Ratio (ICER) and economic model specifications), (B) interpretation/assessment of this evidence (i.e., clinical and economic evidence related uncertainties raised), (C) societal and system-specific context in which HTA-based decision-making operates (i.e., dimensions of value that a technology adds in the society/setting of interest, such as the unmet need it targets in terms of therapeutic treatment availability, the societal benefit it offers in terms of improved patient QoL, functional ability outcomes, all referred to as Social Value Judgements (SVJs)) and system-specific processes for decision-making (e.g., the use of a single or multiple technology appraisal in England), and (D) coverage decision outcome categorised as: (i) L = List (i.e., positive coverage decision), (ii) LWC = List with one or more conditions which are not classified as MEAs (e.g., dosing restrictions, clinical restrictions relating to treatment eligible sub-population, etc.), (iii) LWCMEA = List with one or more conditions including at least one restriction classified as MEA and iv) DNL = Do not list (i.e., negative coverage decision).

Data on the above “buckets”, per medicine-indication pair in all study countries were extracted from publicly available HTA appraisals published in the respective HTA bodies’ websites, namely PBAC (AUS), NICE (ENG), TLV (SE) and SMC (SCOT). A database stratified by HTA agency was built to describe and classify MEAs across the respective HTA bodies and facilitate data analysis.

### Data analysis

Restricted HTA outcomes were coded as a binary variable (i.e., LWC vs. LWCMEA), while the type of MEA was coded as a multinomial variable (i.e., financial (“F”), outcomes (“O”) based or combination (“C”)), based on taxonomies that have been described in the literature [2, 6, 9], with discounts explicitly considered as financial MEAs in this analysis. Finally, assuming that all categories of uncertainties and SVJs were applicable to all drug-indication pairs studied, these were treated and coded as binary variables based on whether they have been raised and considered (or not), respectively, in the HTA-based decision-making process [11]. More specifically, the mention/raise of an uncertainty or SVJ—regardless of its weight/impact on the decision-making process—has been classified as “raised” or “considered”, while in cases where there is no mention of a specific uncertainty or SVJ this was classified as “not raised” or “not considered”, respectively, for each drug-indication pair.

For the purposes of this analysis, a panel data design was not feasible as the study sample comprised one decision per medicine-indication pair per country in a particular year as opposed to annual decisions; similarly, since the response variables are categorical, they could not be modelled as a linear combination of explanatory variables either [15]. Therefore, the associations studied were described as probabilities, estimated by means of a binary and a multinomial logit model. For the first part of the analysis, binary logistic regression was used to estimate the probability of a technology receiving restricted coverage with at least one restriction in the form of a MEA (as opposed to one or more restrictions without a MEA) based on a set of HTA explanatory variables, hypothesised to influence HTA-based decision-making [14, 16]. Additionally, as a robustness check, Pearson’s Chi-squared tests were performed to identify which HTA criteria determine statistically significant differences between LWC and LWCMEA coverage decisions for each study country. Finally, for the second part of the analysis, multinomial logistic regression was used to model the probability of an implemented MEA taking one of the three outcomes “F”, “O” or “C” given a set of HTA criteria/ explanatory variables associated with the medicine-indication pair in question.

## Results

235 coverage decisions were studied, of which 88% (*n* = 207) were favourable (with or without restrictions) and 12% (*n* = 28) non-favourable across all countries. The majority of favourable coverage decisions were LWCMEA (78.7%, *n* = 163), 11.1% (*n* = 23) were LWC and 10.2% (*n* = 21) were L. In England, 93% (n = 54) of MEAs were financial, 96% (*n* = 49) in Scotland, 76.3% (*n* = 29) in Australia and 27% (*n* = 4) in Sweden. Outcome-based schemes were mostly implemented in Sweden (47%, *n* = 7) and combination schemes primarily used in Australia (21%, *n* = 8) (Table 1).

**Table 1:** Study sample characteristics, including, number of favourable decisions (with or without MEA) per country, and the respective types of MEAs implemented (where applicable)

Oncology medicine-indication pairs assessed by the study HTA agencies (*n*, %)					
	England (NICE) (*n* = 68)	Australia (PBAC) (*n* = 64)	Scotland (SMC) (*n* = 61)	Sweden (TLV) (*n* = 42)	All sample (*n* = 235)
List (L)	2 (3%)	1 (1.5%)	4 (6.5%)	14 (33.4%)	21 (8.9%)
List with conditions (LWC)	1 (1.4%)	12 (18.8%)	3 (4.9%)	7 (16.6%)	23 (9.8%)
LWC w/ MEA (LWCMEA) Of which	58 (85.3%)	38 (59.3%)	51 (83.6%)	16 (38%)	163 (69.3%)
Financial	54 (93%)	29 (76.3%)	49 (96%)	4 (27%)	136 (83.4%)
Outcomes	2 (3.5%)	1 (2.6%)	0%	7 (47%)	10 (6.2%)
Combination	2 (3.5%)	8 (21%)	2 (4%)	5 (26%)	17 (10.4%)
Do not list (DNL)	7 (10.3%)	13 (20.3%)	3 (5%)	5 (12%)	28 (12%)

n/a not applicable for the HTA agency of interest, *DNL* do not list, *L* list, *LWC* list with conditions, *LWCMEA* list with conditions including a MEA, *MEA* managed entry agreement, *NICE* National Institute for Health and Care Excellence, *PBAC* Pharmaceutical Benefits Advisory Committee, *SMC* Scottish Medicines Consortium, *TLV* The Dental and Pharmaceutical Benefits Agency

### LWC vs. LWCMEA coverage decision

Of the restricted coverage decisions studied (n = 186), 88% (n = 163) were LWCMEA and 12% (n = 23) were LWC. A number of binary logit models were performed to ascertain the effects of different HTA criteria on the likelihood of receiving a LWCMEA as opposed to a LWC coverage decision. The statistically significant models with the best predictability rate are presented below (Table 2). Values highlighted in bold correspond to the effect size/ likelihood (*i.e*.,  OR) and the respective *p*-value of the HTA criteria that were found to be of statistical significance within the different models.

**Table 2:** Binary logit models, predicting the likelihood (odds ratio (OR)) of a coverage decision being restricted with vs. without MEA, based on the different sets of HTA criteria studied

HTA criteria	Model 1		Model 2		Model 3		Model 4	
	OR	*p*	OR	*p*	OR	*p*	OR	*p*
Clinical uncertainties								
Population generalisability	1.859	.506	2.112	.430	.444	.505	2.352	.219
Study design	1.861	.359	1.977	.319	.535	.464		
Clinical comparator	.585	.459	.594	.477	.835	.361		
Relevance to clinical practice	.415	.241	.493	.349				
Clinical evidence					.292	.589		
Clinical benefit							.791	.688
Economic uncertainties								
Economic modelling	.695	.612	.579	.463	.386	.535	1.264	.672
Cost-effectiveness	**3.926**	**.034**	**4.361**	**.029**	**6.700**	**.010**	**3.240**	**.022**
Utilities	**26.731**	**.018**	**20.970**	**.028**	**7.169**	**.007**		
Model type	1.198	.898	1.954	.653				
Costs	.906	.878	.846	.796				
Economic comparator			13.204	.997	**4.147**	**.042**		
Social Value Judgements								
Rarity	**.147**	**.024**	**.165**	**.040**	3.443	.064	**.254**	**.017**
Special considerations	.553	.452	.478	.359	.361	.548	**3.014**	**.040**
Severity	2.683	.148	2.326	.208	.160	.689		
Unmet need	.905	.880	.834	.789	.003	.956		
Innovation	**8.504**	**.029**	**10.632**	**.026**	**11.727**	**.001**		
Administration advantage	4.721	.160	4.709	.184	2.506	.113		
Impact on society	.292	.356	.259	.316	.035	.852		
Impact on QoL	1.038	.962	.993	.993	.084	.772		
Impact on emotional burden	19.047	.998	16.154	.998				
Impact on functional burden	1.245	.893	.392	.612				
Constant	1.819	.291	1.898	.259	3.667	.000	2.686	.033

Model statistics	*χ*^2^	*p*	*χ*^2^	*p*	*χ*^2^	*p*	*χ*^2^	*p*
Likelihood ratio test	47.659	.000	51.297	.000	18.25	.000	19.45	.003
Hosmer and Lemeshow test	12.348	.136	7.289	.506	.279	.870	5.97	.543
Predictability (%)	92%		91%		87%		87%	

*OR* odds ratio, *p* *p* value, *QoL* quality of life, *χ*^2^ Chi-squared value

The first model (*χ*^*2*^ = 47.7, *p* < 0.0001) explained 43% (Nagelkerke *R*^2^) of the variance in restricted coverage decisions and correctly classified 92% of cases. Medicine-indication pairs with utility and cost-effectiveness related uncertainties were approximately 27 (OR=26.731, *p*<0.05) and 4 (OR=3.926, *p*<0.05) times, respectively, more likely to receive a LWCMEA instead of a LWC coverage decision. The SVJs of innovation and rarity were associated with an increased (OR=8.504, *p*<0.05) and decreased (OR=.147, *p*<0.05) likelihood of a LWCMEA (as opposed to LWC) coverage decision, respectively.

The second model (*χ*^2^ = 51.3, *p* < 0.0001) explained 46% (Nagelkerke R^2^) of the variance in restricted outcomes and correctly classified 91% of cases. Medicine-indication pairs with utility and cost-effectiveness related uncertainties were approximately 21 (OR=20.97, *p*<0.05) and 4.5 (OR=4.361, *p*< 0.05) times respectively, more likely to receive a LWCMEA instead of a LWC coverage decision. The SVJs of innovation and rarity were associated with an increased (OR=10.632, *p*<0.05) and decreased (OR=.165, *p*<0.05) likelihood of a LWCMEA (as opposed to LWC) coverage decision, respectively.

The third model (*χ*^2^ = 18.25 , *p* < 0.0001) explained 30% (Nagelkerke *R*^2^) of the variance in restricted coverage decisions and correctly classified 88% of cases. Medicine-indication pairs with utility and economic comparator related uncertainties were approximately 7 (OR=7.169, *p*<0.01)  and 4 (OR=4.147, *p*<0.05)  times, respectively, more likely to receive a LWCMEA instead of a LWC coverage decision. The SVJ of innovation  was associated with an increased (OR=11.727, *p*<0.01) likelihood of a LWCMEA (as opposed to LWC) coverage decision.

Finally, the fourth model (*χ*^*2*^ = 19.45, *p* < 0.001) explained 19% (Nagelkerke *R*^2^) of the variance in restricted outcomes and correctly classified 87% of cases. Medicine-indication pairs with cost-effectiveness related uncertainties were approximately 3  (OR=3.24, *p*<0.05) times more likely to be classified as LWCMEA instead of LWC. The SVJs of special considerations and rarity were associated with an increased (OR=3.014, *p*<0.05) and decreased (OR=.254, *p*<0.05) likelihood of a LWCMEA (as opposed to LWC) coverage decision, respectively.

### Country-specific outcomes

Pearson’s chi-squared tests were also performed to identify any HTA criteria that determine statistically significant differences between LWC and LWCMEA coverage decisions for each study country. Cost-effectiveness uncertainties determined statistically significant differences between the LWC and LWCMEA groups for England (*χ*^2^ = 8.98, *p* = 0.003), Scotland (*χ*^2^ = 3.97, *p* = 0.046) and Australia (*χ*^2^ = 5.02, *p* = 0.025). Additionally, uncertainties around the economic model used and the utilities included in the model highlighted statistically significant differences between LWC and LWCMEA coverage outcomes in England (*χ*^2^ = 5.65, *p* = 0.017) and Australia (*χ*^2^ = 3.10, p = 0.028), respectively. Finally, uncertainties around clinical evidence  and clinical benefit,  and the SVJ of innovation, underscored statistically significant differences between LWC and LWCMEA groups for Scotland (*χ*^2^ = 3.68, *p* = 0.04), England (*χ*^2^ = 4.98, *p* = 0.026) and Australia (*χ*^2^ = 3.10, *p* = 0.028), respectively (Online resource 1).

### Type of MEA

**Fig. 1:**
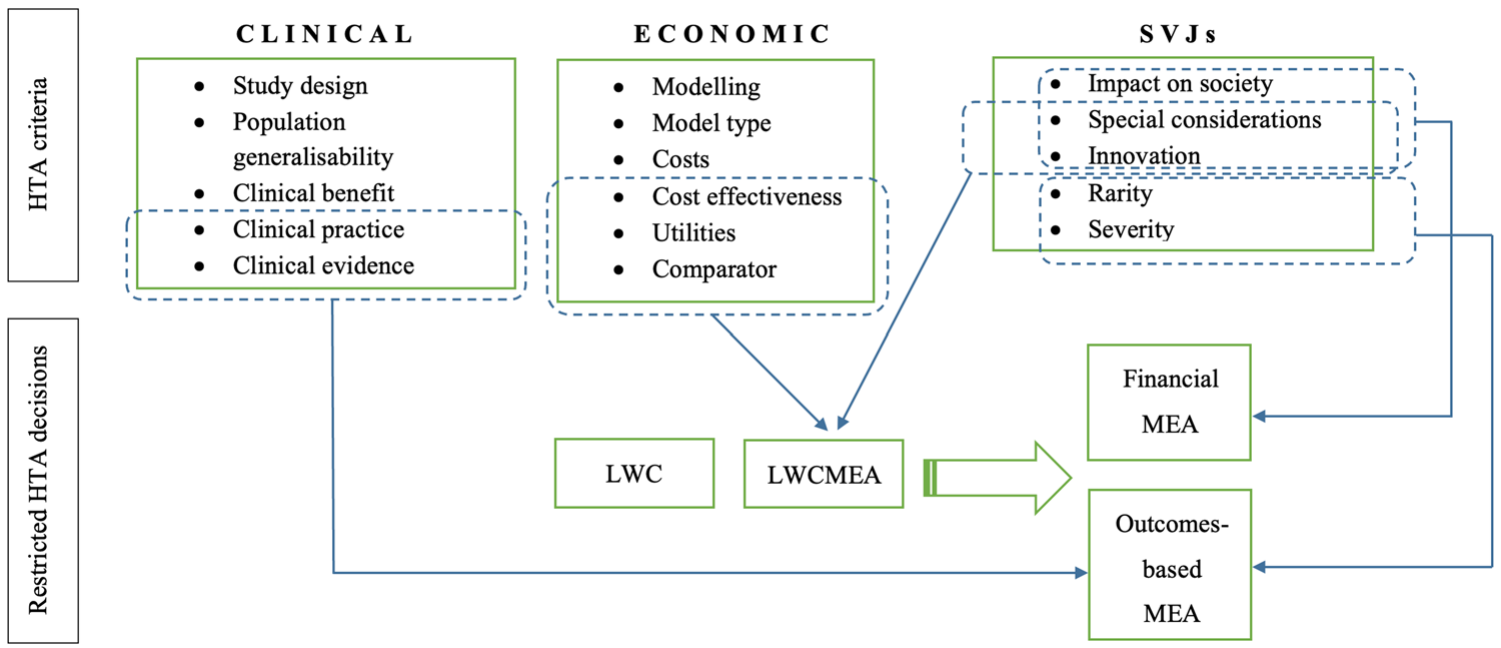
Analytical framework on the HTA criteria driving restricted coverage decisions with a MEA (LWCMEA) and the respective type of MEA. HTA: Health Technology Assessment, LWC: List with conditions, LWCMEA: List with conditions, including a MEA, MEA: Managed Entry Agreement, SVJs: Social Value Judgements. The categories of the HTA criteria included in this analysis and subsequently used in the above framework are based on previously published relevant research and all respective definitions are described in detail therein [11]. Source: The author; the framework is fully conceptualised by the author, based on background from relevant literature [2, 11, 14]

163 MEAs were identified, of which 83.4% (*n* = 136) were “F”, 6.2% (n = 10) were “O” and 10.4% (*n* = 17) were “C”. A number of multinomial logit models were performed to identify the sets of HTA criteria, including clinical/economic uncertainties and SVJs, that best predicted the likelihood of a MEA in place for the study medicine-indication pairs being “F”, “O” or “C” (Table 3, Fig. 1). Values highlighted in bold correspond to the effect size/likelihood (*i.e.*, OR) and the respective p-value of the HTA criteria that were found to be of statistical significance within the different models.

**Table 3:** Multinomial logit models, predicting the likelihood (odds ratio (OR)) and respective statistical significance (*p*) of a MEA being financial or outcomes based or a combination of both, based on the different sets of HTA criteria studied

HTA criteria	Model 1				Model 2			Model 3				
	Financial vs. outcomes^a^		Combination vs. outcomes^a^		Financial vs. outcomes^a^		Combination vs. outcomes^a^	Combination vs. financial^a^			Outcomes vs. financial^a^	
	OR	*p*	OR	*p*	OR	*p*	OR	*p*	OR	*p*	OR	*p*
Clinical evidence	0.097	0.080	0.233	0.332	**0.066**	**0.045**	0.153	0.234	5.262	0.078	**69.221**	**0.023**
Cost-effectiveness	3.033	0.437	0.663	0.800	3.793	0.379	0.691	0.828	0.327	0.207	0.310	0.460
Innovation	**9.346**	**0.042**	0.276	0.412	**8.999**	**0.047**	0.188	0.331	**0.038**	**0.013**	0.072	0.085
Rarity	**0.073**	**0.032**	**0.040**	**0.048**	**0.061**	**0.029**	**0.034**	**0.046**	0.977	0.986	**46.207**	**0.041**
Clinical practice	**0.072**	**0.043**	**0.056**	**0.044**	**0.084**	**0.047**	0.065	0.070	0.786	0.779	**26.400**	**0.046**
Clinical benefit	4.830	.177	16.491	.059	1.000	0.264	1.000	0.110				
Clinical comparator	0.726	0.776	0.627	0.736								
Impact on society					**17.732**	**0.000**	18.416	^b^				
Modelling									0.662	0.641	0.685	0.768
Special considerations									**0.148**	**0.047**	0.139	0.212
Utilities									1.719	0.575	0.257	0.365
Severity									3.490	.278	**23.349**	**0.049**
Intercept		0.011		0.124		0.006		0.056		0.426		0.012

*OR* odds ratio, *p* *p* value

^a^Reference category of the multinomial model

^b^No statistics are computed because variable is a constant

The first model (*χ*^*2*^ = 38.61, *p* < 0.0001) explained 42% (Nagelkerke *R*^2^) of the variance in MEA types. Medicine-indication pairs with uncertainties raised around relevance to clinical practice (OR=.072, *p*< 0.05 and OR=.056, *p*<0.05) and social value considerations around  rarity (OR=.073, *p*<0.05 and OR=.04, *p*<0.05) were more likely to be funded with an “O”, as opposed to a “F” and a “C” agreement respectively. On the contrary, the social value consideration  of innovation  was as associated with an approximately 9.5 (OR=9.346, *p*<0.05) times higher likelihood of a “F” as opposed to an “O” agreement.

The second model (*χ*^*2*^ = 41.79, *p* < 0.0001) explained 45% (Nagelkerke *R*^2^) of the variance in MEA types. Medicine-indication pairs with uncertainties raised around clinical evidence (OR=.066, *p*<0.05) and relevance to clinical practice (OR=.084, *p*<0.05), and social value considerations around rarity  (OR=.061, *p*<.05) were more likely to be funded under an “O” as opposed to a “F” agreement. Similarly, medicine-indication pairs with  social value considerations around  rarity  were  more likely (OR=.034, *p*<0.05) to lead to an “O” as opposed to a “C” agreement. On the contrary, medicine-indication pairs with social value considerations around innovation  and impact on society  were associated with an approximately 9 (OR=8.999, *p*<0.05) and 18 (OR=17.732, *p*<0.0001) times higher likelihood of coverage with a “F” as opposed to an “O” agreement.

The third model (*χ*^*2*^ = 47.94, *p* < 0.0001) explained 50% (Nagelkerke *R*^2^) of the variance in MEA types. Medicine-indication pairs with uncertainties raised around clinical evidence (OR=69.221, *p*<0.05) and relevance to clinical practice (OR=26.4, *p*< 0.05), and social value considerations around rarity (OR=46.207, *p*<0.05) and severity (OR=23.349, *p*<0.05), had a higher likelihood of coverage with an “O” instead of a “F” agreement. Additionally, medicine-indication pairs with social value considerations around innovation (OR=.038, p<0.05) and special considerations (OR=.148, p<0.05)  were associated with a higher likelihood of coverage with a “F” instead of a “C” agreement.

### Discussion and policy implications

This paper explored the sets of HTA criteria, including clinical/economic uncertainties and SVJs, that might contribute to a higher likelihood of restricted HTA recommendations including a MEA as part of the restriction or not, and subsequently identified the HTA-relevant criteria that determine the respective type of a MEA in place (Fig. 1). This is the first study to date to model the HTA criteria that determine both the utilisation and the typology of MEAs in oncology therapies across countries.

Coverage with a MEA was predominantly driven by uncertainties around the utilities of the economic model, and the SVJ of innovation. Outcome-based contracts were primarily influenced by uncertainties on the clinical evidence and relevance to clinical practice, followed by the rarity and severity of the condition. Financial MEAs were influenced by the SVJs of innovation and societal impact of the technology in question. Similar findings arise from the limited and largely descriptive evidence available in the relevant literature. A recent review of outcome-based MEAs in the OECD countries concluded that these may indeed be more common for products with orphan indications, while a case study presented therein concluded that outcome-based schemes in England mostly tried to address uncertainty around the magnitude of long-term clinical benefit, and concerns around the duration of therapy in routine clinical practice [17]. It has also been suggested that outcome-based contracts typically aim to address uncertainties around efficacy or effectiveness in the general population, long-term clinical evidence on clinically significant endpoints (i.e., clinical rather than surrogate markers), as well as safety, and numbers likely to be treated in real-world practice [6]. Finally, using a theoretical model, Antonanzas et al. (2011) analysed situations in which payers will prefer a MEA over non-MEA and concluded that payers’ decisions will depend on monitoring costs, marginal production costs, and the utility patients will derive from treatment [18].

Beyond its empirical study design, another strength of this analysis is the holistic approach considered in the HTA criteria driving MEAs, accounting for the interconnected impact of both uncertainties and SVJs on the final HTA/MEA outcomes, as opposed to the existing literature that has primarily studied the impact of uncertainties. This is important because the emphasis placed on HTA criteria differs between HTA stakeholders across or even within countries; some countries focus on disease severity and drug efficacy, others concentrate on cost-effectiveness, whereas in some countries, payers and HTA stakeholder experts have different preferences on the HTA process and hence, divergent views on which criteria are the most significant within their systems [12]. Specifically, for MEAs, it arises that the requirement for an agreement and the type of agreement preferred by payers, is subject to the disease area and other setting and medicine specific, value considerations [19]. Furthermore, despite significant efforts to create good practice guidelines on the design, implementation, and evaluation of MEAs [6, 20–24], there are still gaps in understanding the conditions that lead to acceptance of proposed MEAs from the payers’ perspective. For example, in England, rejections of manufacturer proposed agreements (i.e., Patient Access Schemes (PAS)) are still observed, highlighting that despite existing guidelines on the submission of PAS, we still lack an understanding of the considerations that render a MEA successful from the point that a company submits a PAS proposal and until this is accepted by NICE [24, 25]. On that front, the findings presented here can enhance transparency in existing guidelines by promoting a shared understanding on the aspects that determine value in MEA negotiations from the payer’s’ perspective. This can guide both manufacturers—to tailor agreement proposals such that they align with the value perceptions of different payers, and payers—to establish more streamlined processes in decision-making under uncertainty.

## Limitations

Despite the empirical contribution of this study in the field of MEA research, the results presented herein should be interpreted with caution due to certain methodological discrepancies that possibly undermine the robustness of the study.

First, country-specific policies, purchasing framework and context in which pricing and reimbursement decision-making operates have not been incorporated in the analysis per se. It is believed that their potential confounding effect has been captured through criteria around HTA system-specific considerations such as SVJs. Of course, even though the SVJ classification used in this analysis is largely applicable to all important SVJs considered across countries [11, 14], SVJs still remain highly subjective and dependent on the setting-specific context in which they are considered. Therefore, the SVJ variables included in this analysis might not be entirely representative of all the system-specific considerations that are of “weight” in HTA-based decision-making across the study countries. In addition, reference in the literature has been made on the impact of the overall country-specific healthcare and welfare characteristics on HTA-based decision-making, such as healthcare spending per capita, societal willingness-to-pay and the structure of the healthcare system [26]. As such, to enhance accuracy of the results, these factors should be explicitly included in future studies modelling the uptake of MEAs.

Second, based on the methodology followed in this analysis, whether an uncertainty has been resolved or not reflects the impact of the implemented MEA, while the mention/raise of an uncertainty during the appraisal (whether resolved or not following the proposed MEA) reflects a potential determinant/reason behind the implementation of a MEA as a funding modality. On that front, this specific analysis does not differentiate between resolved/unresolved uncertainties; it aims to capture all the uncertainties raised (as per the HTA reports/public summary documents) to understand which of these had a greater impact in determining LWCMEA coverage decisions. However, it is of critical importance to conduct further analyses to capture the uncertainties that remain following the proposed MEA, as an evaluation of its impact.

Finally, the limited sample size studied hinders the overall power, sensitivity and specificity of the models. Future replication of these models would benefit from a larger study sample, possibly by including coverage decisions of medicines in other therapeutic areas too, although caution should be exercised to account for potential comparability issues arising from differences in the value that different SVJs reflect for payers in different disease areas. Overall, due to setting-specific nuances in HTA-based and reimbursement decision-making, the criteria and their relative weight in the decision-making process, as identified in this analysis, are not necessarily generalisable across settings and should be interpreted on an individual basis and adapted to the respective setting-specific context in question.

## Conclusions

The growing interest in MEAs and their increased implementation across countries globally, necessitates an enhanced transparency on the aspects that determine value in MEA negotiations. On that front, the findings of this study provide a better understanding on the decision-making criteria that shape payers’ preferences in coverage with a MEA or not. Empirical research on the HTA criteria driving MEAs is key to encourage a transparent, cross-country learning on how MEAs can be tailored to align with payers’ perceptions on “value” and ultimately, promote more efficient MEA negotiations and increased opportunities for coverage through MEAs. There are still barriers to overcome for MEAs to be implemented more widely and efficiently, such as their increased administrative burden, the absence of standardised processes to evaluate their outcomes and the confidentiality around the final prices and discounts negotiated under these agreements.

## Supplementary Information

Below is the link to the electronic supplementary material.

Supplementary file1 (DOCX 23 KB)

## Data Availability

The dataset generated during and/or analysed during the current study are available from the corresponding author on reasonable request.
