# Supplementary material for: Health technology assessment criteria as drivers of coverage with managed entry agreements: a case study of cancer medicines in four countries
Source: Eur J Health Econ. 2022 Oct 11;24(7):1023–31. doi: 10.1007/s10198-022-01526-x (PMC10406668; doi:10.1007/s10198-022-01526-x)
Supplement: Supplementary file 1 — Supplementary file1 (DOCX 23 KB) [file 10198_2022_1526_MOESM1_ESM.docx]

***Title:*** Health Technology Assessment criteria as drivers of coverage with Managed Entry Agreements: a case study of cancer medicines in four countries

**Journal:** *The European Journal of Health Economics*

**Authors and affiliations**

**Olina Efthymiadou, MSc**

Medical Technology Research Group

Department of Health Policy

London School of Economics

Houghton Street

London WC2A 2AE

England

ORCID iD: 0000-0001-9018-7144

**Address for correspondence:**

**Olina Efthymiadou, MSc**

Medical Technology Research Group

Department of Health Policy

London School of Economics

Houghton Street

London WC2A 2AE

England

[a.efthymiadou@lse.ac.uk](mailto:a.efthymiadou@lse.ac.uk)

Online resource 1. Differences in the number (%) of uncertainties and SVJs raised/ considered between LWC and LWCMEA coverage decisions across countries and their respective statistical significance (Pearson’s *x^2^*, *p-value).*

| **HTA criteria** | **England (n=59)** | **Scotland (n=54)** | **Australia (n=50)** | **Sweden (n=23)** |
| --- | --- | --- | --- | --- |
| **Clinical evidence** | 0% vs. 100%  (.69, .40) | **0% vs. 100%**  **(3.68, .04) ^†^** | 26% vs. 74%  (1.18, .27) | 33% vs. 67%  (.059, .80) |
| **Clinical benefit** | **0% vs. 100%**  **(4.98, .026) ^†^** | 3% vs. 97%  (1.38, .240) | 31% vs. 69%  (.099, .75) | 36% vs. 64% (.471, .493) |
| Clinical comparator | 0% vs. 100%  (.605, .437) | 9% vs. 91%  (.329, .566) | 25% vs. 75%  (.005, .94) | 67% vs. 33% (2.139, .144) |
| Study design | 0% vs. 100%  (1.483, .223) | 4% vs. 96%  (.353, .552) | 24% vs. 76%  (.007, .93) | 25% vs. 75% (.068, .795) |
| Population generalisability | 0% vs. 100%  (.983, .321) | 6% vs. 94%  (.021, .885) | 14% vs. 86%  (.460, .49) | 100% vs. 0% (2.39, .122) |
| Clinical practice | 0% vs. 100%  (1.126, .289) | 5% vs. 95%  (.019, .891) | 25% vs.75%  (.002, .96) | 33% vs. 67% (.014, .907) |
| **Modelling** | **0% vs. 100%**  **(5.65, .017) ^†^** | 3% vs. 97%  (2.09, .148) | 24% vs. 76%  (.003, .95) | 44% vs. 56% (1.371, .242) |
| Cost | 0% vs. 100%  (1.59, .207) | 0% vs.100%  (1.00, .316) | 22% vs. 88%  (.167, .68) | 33% vs. 67% (.032, .858) |
| Utilities | 0% vs. 100%  (2.317, .128) | 0% vs. 100%  (1.58, .20) | **0% vs. 100%**  **(3.10, .028) ^†^** | 50% vs. 50% (.396, .529) |
| **Cost-effectiveness** | **0% vs. 100%**  **(8.98, .003) ^†^** | **0% vs. 100%**  **(3.97, .046) ^†^** | **11% vs. 89%**  **(5.02, .025) ^†^** | 37% vs. 63% (1.24, .26) |
| Economic comparator | 0% vs. 100%  (.233, .629) | 0% vs. 100%  (1.724, .189) | 0% vs. 100%  (.676, .411) | 0% vs. 100% (1.509, .219) |
| Rarity | 0% vs. 100%  (.233, .629) | 14% vs. 86%  (1.168, .280) | 37% vs. 63%  (.875, .350) | 50% vs. 50% (1.46, .226) |
| Severity | 0% vs. 100%  (.650, .420) | 4% vs. 96%  (.217, .543) | 25% vs. 75%  (.001, .980) | 25% vs. 75% (2.139, .144) |
| Unmet need | 0% vs. 100%  (2.734, .098) | 7% vs, 93%  (.813, .367) | 25% vs. 75%  (.007, .935) | 36% vs. 64% (.350, .554) |
| **Innovation** | 0% vs. 100%  (2.513, .113) | 5% vs. 95%  (.021, .885) | **0% vs. 100%**  **(3.10, .028) ^†^** | * |
| Administration advantage | 0% vs. 100%  (.233, .629) | 5% vs. 95%  (.021, .885) | 0% vs. 100%  (2.21, .136) | * |
| Impact  on society | 0% vs. 100%  (.115, .734) | 6% vs. 94%  (2.09, .688) | 20% vs. 80%  (.061, .805) | * |
| Impact on QoL | 0% vs. 100%  (.983, .321) | 6% vs. 94%  (.019, .891) | 27% vs. 73%  (.059, .807) | * |
| Special considerations | 0% vs. 100%  (2.734, .098) | 5% vs. 95%  (.227, .634) | 100% vs. 0%  (3.15, .076) | 31% vs. 69% (.002, .968) |
|  |  |  |  |  |
| ***Key:*** *No statistics are computed because variable is a constant.  **^†^** Bold highlighted results denote statistical significance for the respective country in question | | | | |
| ***Note:*** LWC: List With Conditions, LWCMEA: List With Conditions, including a Managed Entry Agreement.  SVJs: Social Value Judgements, QoL: Quality of Life. | | | | |
